# Knowledge and menstrual hygiene practice among adolescent school girls in southern Ethiopia: a cross-sectional study

**DOI:** 10.1186/s12889-019-7973-9

**Published:** 2019-11-29

**Authors:** Zelalem Belayneh, Birhanie Mekuriaw

**Affiliations:** Department of Psychiatry, College of Health and Medical Science, Dilla University, Dilla, Ethiopia

**Keywords:** Menstruation, Hygienic practice, Menses, Cross-sectional study, Adolescents

## Abstract

**Background:**

Menstruation is a normal physiological process of females at their reproductive age. However, it is surrounded with social taboos and supernatural beliefs. The poor knowledge and understanding of menstruation may lead to unsafe hygienic practice that intern increases the risk of reproductive and genito-urinary tract infections, cervical cancer, school drop-out, poor academic performance and overall poor quality of life. Despite such clinical and academic effects, the knowledge and hygienic practice of adolescent girls towards menstruation is not well addressed in Ethiopia, particularly among school adolescent girls. Therefore, the main objective of this study was to assess the knowledge and menstrual hygiene practice among adolescent school girls in southern Ethiopia.

**Methods:**

This was an institutional based cross-sectional study conducted at Gedeo zone high schools among 791 randomly selected adolescent girls using multi stage sampling technique. Data were collected using interviewer administered questionnaire. The collected data were entered to EPI-INFO (soft ware) and exported to SPSS version 20 for analysis. Bivariable and multivariable logistics analyses were computed to identify factors associated with the poor menstrual hygienic practice. During bi-variable analysis, variables with *P*-values of less than 0.25 were entered to multivariable model for further analysis. In the final model, *P*-value of less than 0.05 was used as a base to identify factors having a statistically significant association with poor menstrual hygiene practice at corresponding 95% confidence interval.

**Result:**

From a total of 791 adolescent girls participated in this study, 68.3% had poor knowledge of menstruation. About 48.1% of school girls used absorbent materials, and 69.5% clean their external genitalia. Generally, 60.3% of girls had poor menstrual hygienic practice. Age less than 15 years [OR = 1.71:95% CI (1.22, 2.39)], longer days of menstrual flow [OR = 2.51:95% CI (1.66, 3.80)] and poor knowledge of menses [OR = 1.48:95% CI (1.04, 2.1)] had a significantly associated with poor menstrual hygiene practice.

**Conclusion:**

Majority of adolescent school girls had poor knowledge regarding menstruation and their hygienic practices are incorrect. This demonstrates a need to design acceptable awareness creation and advocacy programs to improve the knowledge and promote safe hygienic practice of adolescent school girls during menstruation.

## Background

Menstruation is a universal and normal phenomenon during the reproductive age of females [1, 2]. The onset of menses takes place during adolescent period in which dominant physiological and emotional changes take place [2, 3]. Adolescent is an essential period where females are preparing and adjusting themselves to manage their menstrual bleeding in safe and clean way [3, 4]. This is also the ideal time that girls often join different environments including high schools and tried to plan for their next adulthood life [3].

However, most adolescent girls (girls with age ranges of 10 to 19 years old) enter to their puberty stage (maturity) without preparing themselves due to the shortage of adequate information [5]. Most women are uncomfortable to discuss regarding “menses” as it is has a social taboo and adolescent girls could not have access to gain adequate information [4, 6]. Even the little information they receive most commonly from religious institutions, peers, family member is often selective and surrounded by misperceptions [7]. For example, people in developing countries like Ethiopia often perceive menstruation as something happened as a result of being cursed, a sign of diseases, punishment from God, a lifelong process and others [7–11]. As a result, adolescent girls perceive menstruation as something embarrassing that should be kept hidden [9–12]. This can increase the vulnerability of adolescent girls to have mental, emotional and physical problems [13–15]. These conditions further impair the daily activities, academic performance, school attendance, and social relationships of adolescent girls [16, 17].

The view in which girls perceive towards menstruation also affects their hygienic practice during their menstrual bleeding [18]. Women with better understanding of menses often have safe and clean way of managing their menstrual bleeding and vice versa [9, 18, 19]. It is uncovered that poor menstrual hygiene practice can be a reason for reproductive and genitor-urinary tract infection, cervical cancer, school absenteeism, or drop-out, poor academic performance, lower self-esteem and poor quality of life [20]. Moreover, girls have also often experienced feelings of fear, confusion and shame during their menstruation period as a result of smell, leakage, staining of clothes and dropping of sanitary materials during their class schedules [21]. This can have also a negative impact on the concentration, class participation and confidence of their studies [22].

Although poor knowledge and unsafe menstrual hygiene practice have such considerable clinical implications for the girls themselves and their future off springs [19], knowledge of adolescent girls regarding menstruation is poor and their hygienic practices are not correct, particularly with lower socio-economic contexts [7, 19]. It has been reported that 40–45% of adolescent school girls have poor knowledge and unsafe hygienic practice of their menstrual bleeding [5]. This might have a clinical implication to integrate the promotion of menstrual hygienic practice in the health care system [21] and comprehensive efforts including policy implication are needed to improve girls’ knowledge and safe hygienic practices towards menstruation right from her adolescent period [21, 22].

Despite safe menstrual hygienic practices can have a paramount importance to help millions of women suffering from such complicated and complex problems [23], it is the missed opportunity to address the level of understanding and hygienic practice of menstruation among girls earlier from their adolescent age in many developing countries, including Ethiopia [24, 25]. Little available studies in Ethiopia had no a particular focus of adolescent school girls’ knowledge and menstrual hygiene practice. Moreover, there is a need to re-examine the association between poor menstrual hygienic practice (PMHP) and obstetric and gynecological factors among adolescent school girls. This is unrecognized and unaddressed public health concern in developing countries like Ethiopia. Therefore, this study was aimed to assess the Knowledge and menstrual hygiene practices of adolescent school girls in Gedeo zone. The result of this study might have a supreme clinical importance and might help for planning, policy preparation and developing appropriate intervention mechanisms. Furthermore, the findings of this study will be used as baseline information for further research works.

## Methods

### Study design, period and setting

This was an institutional based cross sectional study conducted from May 1st/2018 to February 1st/2019 among Gedeo zone high school girls. Gedeo is one of the eleven zones in South Nation, Nationality and Peoples’ Regional (SNNPR) states of Ethiopia. Dilla is the zonal town of Gedeo people located at 359 km south from Addis Ababa (the capital city of Ethiopia). The zone is administrated in to six districts and two town administrations. In the zone, there are about 25 high schools in which a total of 2351 adolescent girls are enrolling.

### Sample size and sampling

We used a single population proportion formula to determine the required sample size for this study. The assumptions made during the sample size calculation was *P* = 39.9% (prevalence of poor knowledge towards menstruation) based on a study done in Jimma [25], 95% confidence interval (CI) and 5% margin of error. By considering a design effect of 2 and 10% non-response rate, the total sample size was 806.

Multi stage sampling technique was used to select study participants. First, six high schools were randomly selected from a total of twenty five high schools found in Gedeo zone. Then, the calculated sample size was proportionally allocated to randomly selected high schools based on the population size. In the second stage, systematic random sampling technique was used in each of randomly selected high schools using a sampling interval (K). The sampling interval (K) was calculated by dividing the total number of eligible adolescent girls to the proportionally allocated samples. Student who had menstrual flow experience and having least two consecutive menstrual cycles within the last 2 months were included so as to minimize recall bias. The first participant was selected between 1 and K using lottery method and “K” value was consequently added until the allocated samples size was addressed.

### Data collection instruments and procedures

Data were collected using structured and interviewer administered questionnaire. The questionnaire was developed from different literatures and contextually adapted to the cultural norms of the study area (Additional file 1). The questionnaire had different section consisting of socio-demographic profile, obstetric and gynecological related characteristics, questions used to assess knowledge of adolescent girls towards menstruation, and questions used to measure hygienic practices of menstruation.

Knowledge of adolescent school girls regarding menstruation was evaluated using a series of 20 Likert scale questions adopted from different literatures [3, 8, 11, 22, 26, 27]. The responses of each 20 item questions had a range of scores from 0 to 3 (0 = strongly disagree, 1 = disagree, 2 = agree, 3 = strongly disagree). The questions had both positively and negatively worded items regarding menstruation, and negatively worded statements were scored reversely. Individuals with a total sum score of 30 (mean score) and above were considered as having a good knowledge regarding menstruation.

The menstrual hygienic practices of adolescent school girls were evaluated using a ten item “Yes” or “No” questions. The response of each item was scored as “1” for correct answers and “0” for false responses. The total sum score of the tool ranges from 0 to 10. The tool has been also used by another study conducted in Jimma University [25] and showed a good internal consistency with Cronbach alpha of 0.87 in this study. Girls with a total sum score of less than 50% (5 points) were considered as having a poor menstrual hygiene practice. The socio-demographic, obstetrics and gynecological and academic related characteristics were also recorded through structured interview.

All contents of the questionnaire was first prepared in English and translated to the local language (Gedeufa) and back to English to check its consistency. The Gedeufa version of the questionnaire was also pretested among 40 adolescent female students which could not be included in the actual data collection to check its understandability and ability to address study objectives. Based on the pretest result, minor modification was done regarding the content of the questionnaire and the expression of some terminologies.

Six data collectors (clinical nurses) and two supervisors (MSc level reproductive health professionals) were participated in the data collection after attending 2 days of training regarding the contents of the questionnaire and the data collection procedures. Before the interview, written consent was obtained from all participants above the age of 16. For minor participants (less than 16 years of age), consent was obtained from the parents/guardians on the behalf of participants under the age of 16 years old after providing a brief explanation about the purpose and objectives of the study. It is already known that menstruation is a sensitive issue surrounded by social taboo and supernatural perceptions. Females, particularly adolescent girls often afraid of discussing topics related menstruation. Therefore, we recruited clinical nurses for data collection by considering that clinical nurse can have better experience to communicate people regarding sensitive topics with therapeutic relationship.

### Operational definitions

An adolescent is defined as a student with age range of 10 to 19 years old.

Early adolescent stands for individuals with age ranges of 10 to 14 years old.

Late adolescent refers to individuals whose age is between 15 and 19 years old.

Homemade absorbents in this study refers to non-commercially made sanitary materials prepared by family members or girls themselves for the purpose of menstrual hygiene practice.

Social taboo refers to the status in which the community wrongly assigns for females with menstrual flow and menstruation itself.

Puberty refers to the developmental stage of adolescents in which they start to show secondary sexual characteristics.

### Data analysis and interpretation

The collected data were first checked for its completeness and consistency. The data then, entered to EPI-info version 3.5 (soft ware) and exported to the Statistical Package for Social Science (SPSS) version 20 for analysis. Descriptive statistics was computed to measure the level of knowledge and hygienic practice towards menstruation. We used bi-variable and multivariable logistic analysis to identify factors associated with the poor menstrual hygiene practice of adolescent school girls. All variables with *P*-values less than 0.25 during bivariable analysis were entered together to a multivariable analysis to control possible cofounders. Accordingly, age, residency, living arrangement, duration of menstrual flow, age at menarche and knowledge regarding menses had *P*-values of less than 0.25 and entered to multivariable analysis. In the final model, lower age, longer duration of menses flow and poor knowledge towards menstruation were found to have statistically significant association with poor menstrual hygiene practice with *P*-values of less than 0.05. The strength of the association was also measured by odds ratio with corresponding 95% confidence interval (CI).

## Results

From a total of 806 adolescent school girls invited to participate, 791 completed the interview with a response rate of 98.1%. The mean (±SD) age of respondents was 16.3 (+ 4.7) years with a minimum and maximum age ranges of 10 and 19 years. More than half, (58.3%) were Gedeo in their ethnicity and 32.2% of adolescent school girls were living with their own parents **(**Table 1**)**.

**Table 1:** Sociodemographic characteristics of adolescent girls at Gedeo zone high schools, Southern Ethiopia 2019 (*n* = 791)

Variables	Categories	Frequency	Percentage
Age	< 15 years	118	14.9
	> 15 years	573	85.1
Living with	Parents	290	31.3
	Relatives	253	27.3
	Peers	215	23.2
Residency	Town	243	30.7
	Rural	548	69.3
Religion	Orthodox	206	26.0
	Protestant	461	58.3
	Muslim	64	8.1
	Others	60	7.6
Ethnicity	Gedeo	409	51.7
	Oromo	182	23.0
	Amhara	133	16.8
	Guragie	25	3.2
	Others	42	5.3
Birth order	First	285	36.0
	In between	326	41.2
	Last	180	22.8
Maternal education	Unable to read and write	278	35.1
	Able to read and write	138	17.4
	Primary	213	26.9
	Secondary	73	9.2
	Diploma and above	89	11.3
Paternal education	Unable to read and write	202	25.5
	Able to read and write	212	26.8
	Primary	271	34.3
	Secondary	44	5.6
	Diploma and above	62	7.8
Family structure	Nuclear	538	68.0
	Extended	253	32.0

### Obstetric and gynecological related factors

Large proportions, 615 (77.7%) of adolescent school girls in Gedeo zone mentioned that they have experienced dysmenorrhea (severe pain during the menstruation) at least once within the last three conscequitave menstrual cycles. More than half, (67.6%) have experienced their menarche (onset of first menses) within the age range of 12–15 years, and 59.7% had irregular menstrual cycles **(**Table 2**).**

**Table 2:** Obstetric and gynecological related characteristics of adolescent girls at Gedeo zone high schools, southern Ethiopia, 2019 (*n* = 791)

Variables	Categories	Frequency	Percentage
Age of menarche	< 12 years	195	24.7
	12–15 years	535	67.6
	> 15 years	61	7.7
Regularity of menses	Irregular	472	59.7
	Regular	319	40.3
Family history of dysmenorrhea	No	509	64.3
	Yes	282	35.7
Duration of menses flow	< 3 days	198	25.0
	3–5 days	503	63.6
	> 5 days	90	11.4
Pain during menstruation	No	171	22.3
	Yes	615	77.7

### Source of information regarding menstruation

About 27.7% of adolescent girls did not have information regarding menstruation and its hygienic practice before their menarche. Of 72.3% school girls having menses related information before the onset of their menses, 38.3% mentioned their mother as the main source of information and 16.3% got such information from their peers. Relatives, teachers, media and others were also mentioned as sources of information related to menstrual bleeding and its safe management by 6.4, 2.4, 7.6 and 1.3% of girls having information, respectively.

### Knowledge of adolescent girls towards menses

There were numerous supernatural and traditional perceptions and beliefs attributed by adolescent school girls towards menstruation. “Disease as a cause of menses flow” and “Menses as a lifelong process” were common misconceptions endorsed by 51.8 and 50.2% adolescent girls, respectively. Generally, 68.3% of adolescent school girls in Gedeo zone had poor knowledge regarding menstrual bleeding (Table 3).

**Table 3:** Knowledge regarding menstrual bleeding among adolescent girls in Gedeo zone high schools, Southern Ethiopia, 2019 (*n* = 791)

Question		Strongly disagree(0)	Disagree(1)	Agree(2)	Strongly agree(3)
Menses					
1	It is a normal phenomena	8.0%	5.9%	10.4%	75.5%
2	It is unique to females	24.7%	17.7%	6.8%	50.8%
3	Life long	3.03%	20.5%	3.2%	46.0%
4	It is a lifelong process	27.1%	16.6%	6.2%	50.2%
5	It will be stopped after initiation of sexual intercourse	31..6%	13.5%	15.3%	18.6%
6	It is a sign of conception	61.8%	11.0%	12.4%	14.8%
7	It has foul smell	13.5%	13.1%	13.8%	59.5%
8	It is pathological	58.9%	8.05	11.8%	21.4%
Sources of bleeding					
9	Uterus	39.1%	52%	23.1%	32.6%
10	Bladder	41.8%	8.3%	18.5%	31.4%
11	Vagina	22.8%	18.5%	11.5%	47.3%
12	Abdomen	40.3%	13.5%	20.5%	25.7%
Causes of menses					
13	Hormonal	33.9%	10.7%	18.6%	36.8%
14	Diseases	51.8%	11.6%	16.3%	20.7%
15	Curse	51.1%	10.5%	8.7%	27.7%
Which is good to be done during menstruation					
16	Not allowed touch others during menstruation	51.1%	11.9%	22.5%	14.5%
17	Not allowed to go to kitchens during menses	53.2%	12.8%	13.7%	20.4%
18	It is not good to discus about menses	50.1%	10.7%	13.3%	25.9%
19	Activities done by menstruating woman are not blessed	39.6%	15.7%	22.4%	224.4%
20	Being free from menses is a fate	37.7%	17.%	22.3%	22.5%

### Hygienic practice of adolescent school girls

Nearly one third, (33.9%) of adolescent school girls did not use sanitary pads during their menstruation period. About 42.4% were using commercially made sanitary pads, and 23.7% used homemade absorbents (dry clothes, double pants, sponges…). More than half, (69.5%) of students clean their external genitalia with water and soap. Generally, 60.3% of girls found to have poor menstrual hygiene practice with 95% CI (56.9, 63.6) **(**Table 4**).**

**Table 4:** Menstrual hygiene practices of adolescents girls at Gedeo zone high schools, Southern Ethiopia, 2019 (*n* = 791)

Practice related questions		Frequency	Percentage
Using absorbent materials during menstruation		523	66.1
Using commercially made sanitary pad during menstruation		336	42.4
Change pads or cloths more than three times a day during menstruation		494	62.4
Using clean clothes with soap and water		151	19.1
Dries cloths in sunlight		375	47.4
Cleans external genitalia during menstruation		517	65.3
Disposes the pads by wrapping with paper		246	30.0
Washes bath daily with soap during menstruation		446	56.4
Cleaning of external genitalia with water and soap during menstruation		550	69.5
Disposes used sanitary pads in dustbin		354	44.7
Over all menstrual hygiene practice	Good	314	39.7
	Poor	477	60.3

### Factors associated with menstrual hygiene practice

Bivariable and multivariable logistic analyses were done to identify factors associated with menstrual hygiene practice. Accordingly, lower age, longer duration of menses flow and poor knowledge towards menstruation had statistically significant association with the poor menstrual hygiene practice **(**Table 5**)**.

**Table 5:** Bivariable and multivariable analysis of factors associated with poor menstrual hygiene practice among adolescent girls at Gedeo zone high schools, Southern Ethiopia, 2019 (*n* = 791)

Variables	Categories	Hygienic practice		COR 95% C1	AOR 95% CI
		Good	Poor		
Age	< 15 years	105	113	1.61(1.18, 2.21)	1.71(1.22, 2.39)**
	> 15 years	209	364	1	1
Residency	Rural	217	331	1.01(0.74,1.38)	1.06(0.77,1.47)
	Town	97	146	1.00	1.00
Living with	Parents	107	146	11	1
	Relatives	96	128	0.97(0.67,1.40)	1.00(0.69,1.47)
	Peers	63	109	1.26(0.85,1.88)	1.29(0.85,1.95)
	Alone	48	94	1.43(0.93,2.2)	1.42(0.912.22)
Duration of menses flow	< 3 days	97	94	1	1
	3–5 days	135	185	1.41(0.98,2.07)	1.37(0.95,1.99)
	> 5 days	82	198	2.49(1.69,3.65)	2.51(1.66,3.80)***
Knowledge towards menses	Good	209	326	1.28(0.92,1.78)	1.48(1.04,2.1)*
	Poor	17	44	2.12(1.13,3.98)	1.64(0.85,1.95)
Age at menarche	< 12 years	17	44	1.22(0.90,1.66)	1.05(0.76,1.45)
	12–15 years	209	326	1.34(0.71, 2.43)	1.12(0.13,2.34
	> 15 years	88	107	1	1

*P-value < 0.05, **P < 0.01, ***P < 0.001

## Discussion

Adolescence is recognized as a special critical period of females in which significant hormonal and emotional changes take place including their first menstrual onset [5]. Although menstruation is such a normal physiological process in females reproductive age, it is surrounded by taboos and supernatural perceptions [11]. As a result, many adolescent girls could not have the access to get adequate information regarding menstruation and its hygienic practice, and they often join to their menarche without preparing themselves, particularly in rural areas [8]. This might result in adverse health outcomes and poor academic performance of adolescent school girls [3].

In the current study, 68.3% of adolescent school girls in Gedeo zone had poor knowledge regarding the bleedings of menses. This is in line with other similar studies done in Southern Ethiopia (60.1%) [25] and Nigeria which states that perceptions of menstruation are poor and practices are often unsafe among adolescent school girls in Nigeria [9]. However, the finding of the current study showed a better knowledge towards menstruation among adolescent school girls as compared to a similar study done in India, which revealed that 71.3% of female students had poor knowledge regarding their menstruation [8]. The possible explanation for this discrepancy might be the measurement techniques studies used to assess level of knowledge and the socio-cultural differences of study participants.

Regarding hygienic practices of menstrual bleeding, this study showed that nearly half (33.9%) of girls did not use sanitary pads during menstruation and 60.3% of adolescent school girls were identified as having a poor menstrual hygiene practice in general. This is supported by other studies conducted in Northwest Ethiopia [26] and Tehran district [22] stating that adolescent girls had different traditional and supernatural perceptions regarding menstruation and their hygienic practice was not safe. The finding of this study is also in line with findings of studies of western Ethiopia (60.1%) [27], Nigeria (55.7%) [9] and India (64.0%) [8]. This can have a clinical implication for the development and adaptations of contextualized and culturally validated measurement techniques and interventional approaches for adolescent school girls. Moreover, integration of menstrual hygiene promotion in different health care systems and female clubs in schools is also recommended.

However, studies conducted in Persian [21] and Kuwait [17] showed better hygienic practices of adolescent school girls towards menstruation than the finding of the current study. This is possibly explained by the fact that girls in Ethiopia are more attributed by supernatural and cultural perceptions and beliefs regarding menstruation that may restrict them to discuss topics regarding menses and hinder their safe hygienic managements. The other possible explanation for the discrepancy of this result might be due to the economical constraints of Ethiopian adolescent girls to buy commercially made sanitary pads needed for menstrual hygiene practice [4]. This demonstrates a need to provide or show means to access sanitary pads for adolescent school girls with economical constraints.

The second objective of this study was to identify factors associated with hygiene practices of adolescent school girls. Accordingly, the odds of having poor menstrual hygiene practice among adolescent school girls with longer duration of menstrual flow were 2.51 times higher as compared to girls with shorter duration of menstrual flow. The possible explanation for this association might be due to the fact that girls with longer duration of menstrual flow may have economical constraints to buy commercially made absorbents for longer time [26]. In addition, the longer duration of menstrual flow may affect the psychological and emotional states of girls which may further diminish their motivation and commitment to perform safe hygienic practices [22].

It is investigated that knowledge towards menstruation have significant associations with menstrual hygienic practices of girls [27–29]. The findings from the current study also confirmed this explanation by showing that girls with poor knowledge regarding menstrual flow were 1.48 more likely to practice their menstrual hygiene incorrectly. This is possibly due to the effects of cultural beliefs and social taboo regarding menstruation and its hygienic practices attributed by the community they live [19, 30, 31].

Similarly, the odds of poor menstrual hygiene practice among adolescent school girls with age less than 15 years were increased by 1.71 times as compared to girls whose age was equal or greater than 15 years. This is possibly explained by the fact that girls with elder age can have a better opportunity to share more information, gain adequate knowledge regarding menstrual hygiene, and prepare themselves to demonstrate safe hygienic practice during their menstruation period as compared to girls with lower ages [31, 32].

### Strength and limitations of the study

This study was conducted using intensive and culturally adapted tools. The study also tried to evaluate the knowledge and hygienic practices regarding menstruation in a private circumstance by recruiting female data collectors to minimize social desirability bias. However, this study has limitation. First, the cross-sectional nature of the study design might not show the cause and effect relationships between study variables. Second, this study follows only quantitative data collection, and it is not triangulated by mixed approaches. Therefore, further longitudinal and mixed approach study design with more exhaustive and mutually exclusive categories of variables is recommended.

## Conclusions

Majority of adolescent school girls had poor knowledge regarding menstruation and their hygienic practices are incorrect. Lower age, longer duration of menses flow and poor knowledge towards menstruation were significant correlates of poor hygienic practice. This demonstrates a need to design acceptable awareness creation and advocacy programs for adolescent school girls and the public to improve the knowledge and safe hygienic practice of their menstruation flow.

## Supplementary information

**Additional file 1.** Interview guide used for the data collection of this study.

## Data Availability

All data and materials of this study is available and can be accessed from Zelalem Belayneh (corresponding author) with the email address of “zelalembe45@gmail.com”.

## Abbreviations

AOR: Adjusted Odds Ratio
CI: Confidence Interval
COR: Crude Odds Ratio
MHP: Menstrual Hygiene Practice
MSc: Masters of Science
PMHP: Poor Menstrual Hygiene Practice
RTI: Reproductive Tract Infections
SD: Standard Deviation
SPSS: Statistical Packages for Social Sciences
