# Supplementary material for: Knowledge and menstrual hygiene practice among adolescent school girls in southern Ethiopia: a cross-sectional study
Source: BMC Public Health. 2019 Nov 29;19:1595. doi: 10.1186/s12889-019-7973-9 (PMC6884885; doi:10.1186/s12889-019-7973-9)
Supplement: Supplementary file 1 — Additional file 1. Interview guide used for the data collection of this study. [file 12889_2019_7973_MOESM1_ESM.docx]

**Additional files**

**Additional file one**

**Interview guide used for the data collection of this study**

**Part one**

**Socio-demographic characteristics related questions**

| **Questions** | **Response** |
| --- | --- |
| **What is your age in years?** | --------------------------------- |
| **With whom are you living with?** | 1. Parents 2. Relatives 3.Peers 4. Alone   5. Others ---------- |
| **Where is your permanent residence?** | 1. Town 2. Rural |
| **What is your religion?** | 1. Orthodox 2. Protestant 3. Muslim 2. Others---------- |
| **What is you ethnicity?** | 1. Gedeo 2. Oromo 3. Amhara 4.Guragie 2. Others--------- |
| **What is your birth order with your brothers and sisters?** | 1. First 2. In between 3. Last |
| **What is the educational level of your mother?** | 1. Unable to read and write 2. Able to read and write 3. Primary 4. Secondary 5. Diploma and above |
| **What is the educational level of your father?** | 1. Unable to read and write 2. Able to read and write 3. Primary 4. Secondary 5. Diploma and above |
| **What is your family structure?** | 1. Nuclear 2. Extended |

**Part two**

**Obstetric and gynecological related factors**

| **Questions** | **Responses** |
| --- | --- |
| At what age have you experienced menstrual bleeding for the first time? | While I was--------------- years old. |
| Was your menstrual bleeding available for every month for the last six consecutive menstrual cycles? | 1. Yes 2. No |
| Do you have any family history of dysmenorrhea (sever pain during menstrual flow)? | 1. Yes 2. No |
| For how may day dose your menstrual bleeding stayed? | 1. <3 days 2. 3-5 days 3. >5 days |
| Do you have severe pain during your menstrual flow (dysmenorrhea)? | 1. Yes 2. No |

**Part three**

**Source of information and knowledge related questions regarding to menses**

1. Have you ever heard any information regarding menses and its hygienic practice before the onset of your first menstrual bleeding? 1. Yes 2. No
2. If you answered “Yes” for the above question, which of the following was/were your sources of information? 1. Mother 2.Relatives 3. Teachers 4. Media 5. Others ---------

**Please select your appropriate response regarding menses from the given alternatives**

| **Question** | | **Possible options** | | | | | | | |
| --- | --- | --- | --- | --- | --- | --- | --- | --- | --- |
|  |  | Strongly disagree(0) | | | Disagree(1) | Agree(2) | | | Strongly agree(3) |
| 1 | It is a normal phenomena |  | | |  | |  |  | |
| 2 | It is unique to females |  | | |  | |  |  | |
| 3 | It is a lifelong process |  | | |  | |  |  | |
| 4 | It will be stopped after initiation of sexual intercourse |  | | |  | |  |  | |
| 5 | It is a sign of conception |  | | |  | |  |  | |
| 6 | It has foul smell |  | | |  | |  |  | |
| 7 | It is a pathological condition |  | | |  | |  |  | |
| What do you think about the sources of menstrual bleeding? | | | 1. Uterus 2. Bladder 3. Vagina 2. Abdomen 4. I do not know | | | | | | |
| What is the cause of menstrual bleeding according to your perception? | | | 1. Hormonal 2. Diseases 3. Curse 2. Others 5. I do not know. | | | | | | |
| **What do you perceive about the following questions regarding menstruation? Please say “Yes” for correct statements and ‘ no” for false statements** | | | | | | | | | |
| It is not allowed touch others during menstruation | | | | 1. Yes 2. No | | | | | |
| It is not allowed to go to kitchens during menses | | | | 1. Yes 2. No | | | | | |
| It is embarrassing/not good/ to discuss with someone about menses | | | | 1. Yes 2. No | | | | | |
| Activities done by menstruating woman are not blessed | | | | 1. Yes 2. No | | | | | |
| Being free from menses is a fate | | | | 1. Yes 2. No | | | | | |

**Part four**

**Questions related hygienic practice of adolescent school girls during their menstrual flow**

**Please answer the following question by saying “Yes” for activities that you commonly practice during menstrual practice and “No” for activities that you are not practicing yet.**

| I always use absorbent materials during my menstrual flow | 1. Yes 2. No |
| --- | --- |
| I always use commercially made sanitary pad during my menstruation period | 1. Yes 2. No |
| I change pads or cloths more than three times a day during menstruation | 1. Yes 2. No |
| I use clean clothes and wash with soap and water during my menstrual bleeding | 1. Yes 2. No |
| I commonly dry sanitary clothes with sunlight | 1. Yes 2. No |
| I frequently clean my external genitalia during menstruation | 1. Yes 2. No |
| I dispose the pads by wrapping with paper | 1. Yes 2. No |
| I wash bathes daily with soap during menstruation | 1. Yes 2. No |
| I always clean external genitalia with water and soap during menstruation | 1. Yes 2. No |
| I dispose used sanitary pads in dustbin | 1. Yes 2. No |
